# Traffic accident mortality in Najafabad, Iran: a time series model

**Published:** 2019-08

**Authors:** Moslem Taheri Soodejani, Marzieh Mahmoodimanesh, Leili Abedi, Azimeh Ghaderi

**Affiliations:** ^*a*^Social Determinants of Health Research Center, Institute for Futures Studies in Health, Kerman University of Medical Sciences, Kerman, Iran.; ^*b*^Department of Epidemiology and Biostatistics, School of Health, Kerman University of Medical Sciences, Kerman, Iran.; ^*c*^Department of Statistics and Epidemiology, Faculty of Health, Kerman University of Medical Sciences, Kerman, Iran.; ^*d*^Department of fighting against Disease Najaf Abad Health Services center, Esfahan University of Medical Sciences, Najaf Abad, Iran.

## Abstract

**Background:**

Road traffic accidents and their related deaths have become a major concern in Iran. Based on estimates, Iranian road traffic accidents lead to about 30,000 deaths annually. Objectives: In this study, we used a time series model to understand the trend of accidents, and ascertain the viability of applying ARIMA models on data from Najafabad, Iran.

**Methods:**

This study is a cross-sectional study. We used data from accidents occurring in Najafabad between 2011 and 2017. We used the time series method for determining the trend and forecasting. Non-stationary data in mean and variance were removed using Box-Cox transformation. Autocorrelation function (ACF) and partial autocorrelation function (PACF) plots were used for identifying the models which fit data. All analyses were performed using the Minitab 17.

**Results:**

The result of the trend analysis illustration showed a descending trend of the fatalities due to traffic accidents. The highest values of fatalities have occurred in 2011 (97cases). Also, the lowest values of fatalities have occurred in 2014 with 50.51% reduction in comparison to 2011. The ARIMA (0, 1, 1) model was identified as the best-fit model for data. Prediction values of traffic accident fatalities showed a decreasing trend in deaths in the coming years.

**Conclusions:**

Applying this information can be useful to policymakers and managers for planning and implementing special interventions to prevent and limit future accidental deaths.

**Keywords:**

Road Traffic Accidents, Mortality, Time series, Trend

